# Cytokines and Chemokines in Pediatric Appendicitis: A Multiplex Analysis of Inflammatory Protein Mediators

**DOI:** 10.1155/2019/2359681

**Published:** 2019-02-21

**Authors:** S. Ali Naqvi, Graham C. Thompson, Ari R. Joffe, Jaime Blackwood, Dori-Ann Martin, Mary Brindle, Herman W. Barkema, Craig N. Jenne

**Affiliations:** ^1^Department of Community Health Sciences, Cumming School of Medicine, University of Calgary, Calgary AB, Canada; ^2^Department of Production Animal Health, Faculty of Veterinary Medicine, University of Calgary, Calgary AB, Canada; ^3^Department of Pediatrics, Cumming School of Medicine, University of Calgary, Calgary AB, Canada; ^4^Department of Emergency Medicine, Cumming School of Medicine, University of Calgary, Calgary AB, Canada; ^5^Department of Pediatrics, Division of Critical Care, University of Alberta, Edmonton AB, Canada; ^6^Department of Pediatrics, Division of Critical Care Medicine, Cumming School of Medicine, University of Calgary, Calgary AB, Canada; ^7^Department of Surgery, Cumming School of Medicine, University of Calgary, Calgary AB, Canada; ^8^Department of Microbiology, Immunology and Infectious Diseases, Cumming School of Medicine, University of Calgary, Calgary AB, Canada; ^9^Department of Critical Care Medicine, Cumming School of Medicine, University of Calgary, Calgary AB, Canada

## Abstract

**Objectives:**

We aimed to demonstrate the potential of precision medicine to describe the inflammatory landscape present in children with suspected appendicitis. Our primary objective was to determine levels of seven inflammatory protein mediators previously associated with intra-abdominal inflammation (C-reactive protein—CRP, procalcitonin—PCT, interleukin-6 (IL), IL-8, IL-10, monocyte chemoattractant protein-1—MCP-1, and serum amyloid A—SAA) in a cohort of children with suspected appendicitis. Subsequently, using a multiplex proteomics approach, we examined an expansive array of novel candidate cytokine and chemokines within this population.

**Methods:**

We performed a secondary analysis of targeted proteomics data from Alberta Sepsis Network studies. Plasma mediator levels, analyzed by Luminex multiplex assays, were evaluated in children aged 5-17 years with nonappendicitis abdominal pain (NAAP), acute appendicitis (AA), and nonappendicitis sepsis (NAS). We used multivariate regression analysis to evaluate the seven target proteins, followed by decision tree and heat mapping analyses for all proteins evaluated.

**Results:**

185 children were included: 83 with NAAP, 79 AA, and 23 NAS. Plasma levels of IL-6, CRP, MCP-1, PCT, and SAA were significantly different in children with AA compared to those with NAAP (*p* < 0.001). Expansive proteomic analysis demonstrated 6 patterns in inflammatory mediator profiles based on severity of illness. A decision tree incorporating the proteins CRP, ferritin, SAA, regulated on activation normal T-cell expressed and secreted (RANTES), monokine induced by gamma interferon (MIG), and PCT demonstrated excellent specificity (0.920) and negative predictive value (0.882) for children with appendicitis.

**Conclusions:**

Multiplex proteomic analyses described the inflammatory landscape of children presenting to the ED with suspected appendicitis. We have demonstrated the feasibility of this approach to identify potential novel candidate cytokines/chemokine patterns associated with a specific illness (appendicitis) amongst those with a broad ED presentation (abdominal pain). This approach can be modelled for future research initiatives in pediatric emergency medicine.

## 1. Introduction

Appendicitis results in both local and systemic inflammatory changes, which often clinically manifest with right lower quadrant (RLQ) abdominal pain, fever, nausea/vomiting, and anorexia [1] and, left untreated, can progress over the course of the illness to peritonitis, abscess formation, sepsis, and death [2–4]. Not surprisingly, clinicians take advantage of this inflammatory landscape by including laboratory markers as part of the standard workup of children presenting to the Emergency Department (ED) with abdominal pain and suspected appendicitis; most commonly, this includes white blood cell count (WBC), neutrophil count (NC), C-reactive protein (CRP), and/or procalcitonin (PCT) [5]. While elevated levels of such markers certainly help to *support* a clinical suspicion, their individual test characteristics (sensitivity, specificity, and predictive values) are suboptimal for use as diagnostic tests.

Attempts to identify novel appendicitis-specific biomarkers have significantly increased over the last decade. Interleukins (IL) 6 [6–11] and 10 [6, 11, 12] have been the subject of multiple recent studies, as has serum amyloid A (SAA) [13, 14]. While offering some promise, the overall accuracy of these tests remains to be determined. Furthermore, the majority of attempts to identify appendicitis-specific biomarkers have focused on individual proteins. Given the diverse etiological causes of abdominal pain in children, it is unlikely that a single biomarker will definitively identify those children with true appendicitis from those with alternate causes of intra-abdominal inflammation (mesenteric adenitis, viral gastroenteritis, inflammatory bowel disease, etc.); it is more likely that a *combination* of protein mediators will separate different etiologies, using multiple data elements similar to an inflammatory “fingerprint.”

In this study, we demonstrate the potential of precision medicine to describe the inflammatory landscape present in children with appendicitis. Our primary objective was to compare levels of individual inflammatory protein mediators previously associated with intra-abdominal inflammation (CRP [7–11, 15–21], PCT [19–25], interleukin-6 (IL-6) [6–11], IL-8 [6, 7, 17, 26], IL-10 [6, 11, 12], and monocyte chemoattractant protein-1 (MCP-1) [6, 13], SAA [13, 14, 27–29]) in a cohort of children with suspected appendicitis. Furthermore, using a targeted multiplex proteomics approach, we examined an expansive array of novel candidate cytokine and chemokines within this population. Using suspected appendicitis as a high-volume, high-stakes model, we aim to show how precision medicine profiling could be applied across a number of pediatric emergency medicine (PEM) presentations to shape the next generation of PEM research initiatives.

## 2. Methods

### 2.1. Study Design

We completed an observational multicohort study. Data for the current analysis are a subset of the data collected as part of a series of studies through the Alberta Sepsis Network assessing inflammatory protein mediators in children with infection. These studies were approved by the Health Research Ethics Board of the University of Alberta and the Conjoint Health Research Ethics Board of the University of Calgary (REB13-0586; REB15-1045; Pro00008797). Informed consent or assent was obtained from the children and/or their caregivers. In those circumstances where ongoing resuscitative measures were underway, delayed consent was obtained at the earliest possible opportunity.

### 2.2. Study Setting and Population

Study participants were enrolled at the Alberta Children's Hospital, the tertiary pediatric health centre serving southern Alberta, eastern British Columbia, and western Saskatchewan (catchment 1.8 million).

Between 2009 and 2015, 3 cohorts of children were prospectively enrolled according to the inclusion and exclusion criteria described below.

1. Suspected appendicitis: children presenting to the ED with abdominal pain in whom the managing physician suspected a diagnosis of appendicitis defined by either (a) performance of an ultrasound (US) evaluation of the appendix or (b) consultation with the pediatric surgical team for suspected appendicitis. Children were excluded if they had previous appendectomy, required active resuscitation in the ED, were discharged directly to the PICU, were pregnant, had abdominal pain for more than 5 days, had a history of illness resulting in immune suppression, were previously enrolled in the study, or had an imaging study of the appendix performed at an external healthcare centre [30, 31].
2. Sepsis: children presenting to the ED with sepsis, defined as SIRS caused by a suspected or proven bacterial or fungal infection and who had antibiotic/antifungal medications and blood culture ordered, but did not require PICU care [32].
3. Severe sepsis: children admitted to the pediatric intensive care unit (PICU) for sepsis as evidenced by the systemic inflammatory response syndrome (SIRS) caused by a suspected or proven bacterial or fungal infection orders for antibiotics/antifungal medication and an arterial and/or central venous line were required. Children were excluded if they were not expected to survive ≥24 hours, had palliative goals of care (no intubation or vasoactive infusions), or had severe sepsis for ≥48 hours (defined as sepsis with cardiovascular dysfunction, acute respiratory distress syndrome, or two other organ dysfunctions) [32]

For the purposes of the current analysis, we limited inclusion to those children aged 5 through 17 years, as these are the most typical ages of appendicitis presentations. We excluded children from these studies that were enrolled external to the Alberta Children's Hospital. Those children that were co-enrolled in more than one of the cohorts were analyzed once.

### 2.3. Sample Collection and Preparation

Methods for sample collection and preparation were performed as previously described [30, 31, 33]. Briefly, blood samples were obtained from an in situ peripheral intravenous/central line or concurrent with clinically indicated phlebotomy. Prior to receiving any antimicrobial medication, 2 mL (age 5-14 years) to 4 mL (age > 14 years) of whole blood was collected into a heparinized plasma vacutainer tube. Immediately after collection, blood samples were gently inverted several times, placed on ice. Plasma was separated through centrifugation of the tubes at 1200 g for 10 min at 4°C in a swinging bucket centrifuge. Plasma was carefully transferred to a 4 mL cryovial and immediately stored at -70°C. Samples underwent one extra freeze-thaw cycle for aliquoting and distribution for batched analysis. Processing was performed by CCEPTR (Critical Care Epidemiologic and biologic Tissue Resource, a critical care tissue bank at the University of Calgary).

### 2.4. Care of the Patient

Clinical care of the child was left to the discretion of the managing ED and surgical teams according to our local appendicitis and/or sepsis pathways.

### 2.5. Inflammatory Protein Mediator Profiling

The laboratory technicians performing the analyses were blinded to patient allocation. Inflammatory mediators were measured according to standardized, validated processes as previously described [30, 31, 33]. Three human cytokine and chemokine assay kits (Bio-Plex Pro Human Cytokine 21-Plex Assay, Bio-Plex Pro Human Cytokine 27-Plex Assay, Bio-Plex Pro Human Acute Phase 5- + 4-Plex Panel Complete Kit), obtained from Bio-Rad Laboratories Inc. (Hercules, CA, USA), were used to detect 57 inflammatory mediators in the plasma samples (Supplementary Table 1). Assays were run according to manufacturer-provided protocols. Briefly, analyte capture beads were sonicated to disrupt and eliminate any aggregates. Once the beads had been sonicated, 50 *μ*L of the bead mix was aliquoted into each assay well of a 96-well microtiter plate. Beads were washed twice with supplied wash buffer using a magnetic plate base. Standards, controls, or samples were added to each well and incubated for 30 min at room temperature on a horizontal shaking platform. Bead-analyte complexes were washed three times followed by the addition of 25 *μ*L of biotinylated detection antibody to each well. Plates were incubated while shaking for 30 min at room temperature. Bead-analyte complexes washed three times and 50 *μ*L of phycoerythrin- (PE-) conjugated streptavidin was added to each well. Plates were incubated while shaking for 10 min at room temperature and washed three times, after which beads were resuspended in 125 mL of assay buffer and analyzed on a Luminex 200 apparatus (Applied Cytometry Systems, Sheffield, UK). Acquisition and analysis of the samples were driven by Bio-Plex Manager 6.0 software (Bio-Rad Laboratories Inc.). If the coefficient of variance between two replicates was greater than 20%, the data was considered as a missing value. Individual data points that were below the limit of detection were set to the lowest observed value for that protein. Similarly, individual data points that were above the limit of detection were set to the highest observed value for that protein. Some mediators measured were consistently outside of the dynamic range of the assay. Any mediator with >40% of samples analyzed falling outside of the detectable limits were excluded from the overall analysis.

### 2.6. Measures

The primary outcome evaluated was the presence of appendicitis (yes/no), defined as (a) the presence of an inflamed appendix on pathological evaluation or (b) management of appendicitis via percutaneous drain + antimicrobials for an appendiceal abscess. Those children who were evaluated for suspected appendicitis but did not undergo surgical intervention were considered not to have appendicitis provided they did not return for appendectomy within 2 weeks of the index ED presentation. At the time of the studies, nonoperative management of appendicitis was not offered at the study site.

Secondary outcomes included severity of disease. Simple appendicitis included those children with a diagnosis of appendicitis (as above) without evidence of perforation. Perforated appendicitis was identified by evidence of inflamed appendix along with any presence of perforation on pathologic examination or percutaneous management for an appendiceal abscess. Septic appendicitis was defined as a those children with appendicitis admitted to the PICU specifically as a result of the infectious process.

### 2.7. Statistical Analysis

All statistical analyses were conducted in R v.3.4.2 (R Core Team, 2017), and a *p* value <0.05 was considered statistically significant.

### 2.8. Regression Models

Two different groupings of patients were used in the analysis of seven biomarkers chosen a priori, to compare biomarker concentrations between patients with and without appendicitis, and to compare between patients of different severities of appendicitis. For the first analysis, all patients with appendicitis, perforated appendix, and appendicitis causing sepsis were grouped together into the “Appendicitis” category and compared against patients without appendicitis and those with nonappendicitis sepsis. For the second analysis, patients were separated into four categories: patients without appendicitis, patients with appendicitis, patients with severe appendicitis (perforated appendix and appendicitis causing sepsis grouped together due to sample size in each group), and patients with nonappendicitis sepsis.

Biomarker concentrations were transformed using the natural logarithm prior to analysis to normalize the data, and differences in mean log concentrations of the seven biomarkers chosen a priori were assessed using multivariate normal regression models. Multivariate regression models take into account within-patient correlations in the concentrations of different biomarkers and adjust the coefficient and standard error estimates to reflect that. In addition to patient category, age and sex of the patient were also included in the model, to adjust for any age or sex-specific differences in concentrations and provide population-averaged estimates. The same type of model was used for the appendicitis versus no-appendicitis analysis as with the appendicitis severity analysis. After fitting the models, pairwise comparisons between patient categories (averaged over age and sex) were conducted using least-square means predictions and the Bonferroni correction for multiple comparisons through the R package “emmeans” [34].

### 2.9. Decision Tree

Prior to analysis using a decision tree model, proportions of missing observations were determined for all biomarkers, and any biomarker with 15% or more missing values were excluded from the analysis. For all the included biomarkers, missing values were replaced with the mean concentration across all patient categories to minimize bias. The patient categories used for these predictions were the same as the appendicitis severity regression models.

The decision tree was fit using the R package “rpart” [35] on the entire dataset at once as opposed to having separate training and validation datasets. An initial tree using all biomarkers was fit, after which a plot of the error rate as a function of the complexity parameter was created. The complexity parameter represents the number of splits in the decision tree and reflects the number of biomarkers used in the prediction of the outcome. Based on the results of this plot, the complexity parameter at which error rate stops decreasing was identified, and the tree was pruned to this level by removing uninformative biomarkers. The resulting decision tree includes only biomarkers which were identified to be informative and most important for reducing misclassification errors.

## 3. Results

**Figure 1:**
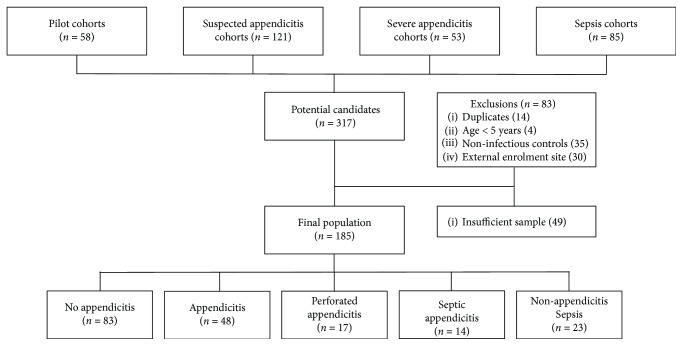
Selection of study candidates and final illness categories.

There were 317 potentially eligible participants in the parent cohorts, of which 83 were excluded. A further 49 subjects did not have adequate sample for testing, leaving a final population of 185 children (Figure 1). Overall, 79 children were included in the appendicitis cohort, 83 in the nonappendicitis cohort, and 23 in the nonappendicitis sepsis cohort. Patient characteristics are outlined in Table 1. Of the 57 inflammatory protein mediators evaluated, 3 (SCGF-B, fibrinogen, haptoglobin) consistently demonstrated values outside of reference range and were excluded from further analysis.

### 3.1. Appendicitis vs Nonappendicitis Abdominal Pain vs Nonappendicitis Sepsis

**Figure 2:**
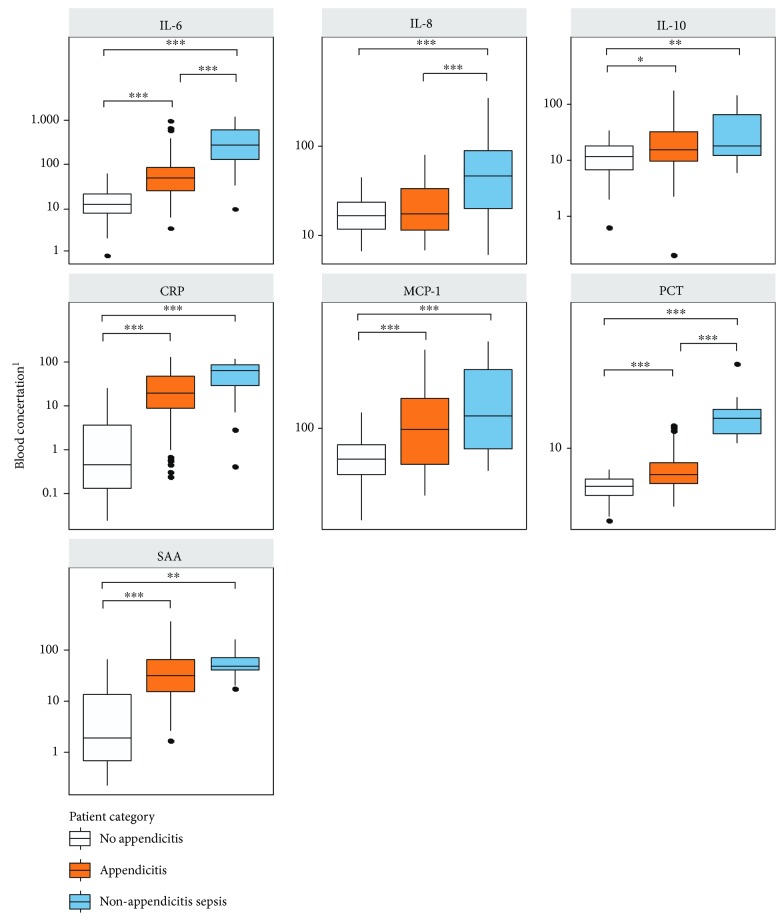
Boxplots of 7 selected cytokine concentrations in pediatric patients (*n* = 196) grouped by category. Outliers (values >95^th^ percentile) are not presented but were included in statistical comparisons using multivariate normal regression followed by least-square means post hoc pairwise contrasts using the Bonferroni correction [1]. Concentrations are in ng/mL for PCT, mg/L for CRP and SAA, and pg/mL for IL-6, IL-8, IL-10, and MCP-1, ^∗^ *p* value <0.05, ^∗∗^ *p* value <0.01, and ^∗∗∗^ *p* value <0.001.

Plasma levels of IL-6, CRP, MCP-1, PCT, and SAA were significantly different (*p* < 0.001) in children with appendicitis compared to those with nonappendicitis abdominal pain. Similarly, significant differences in IL-6, IL-8, and PCT were demonstrated when comparing children with appendicitis and those with nonappendicitis sepsis. IL-6 and PCT were the only markers demonstrating significant differences between all 3 distinct populations (Table 2, Figure 2). Pairwise Pearson's correlation coefficients and multivariate regression model coefficients are listed in Supplementary Tables 2 and 3.

### 3.2. Appendicitis Severity Assessment

**Figure 3:**
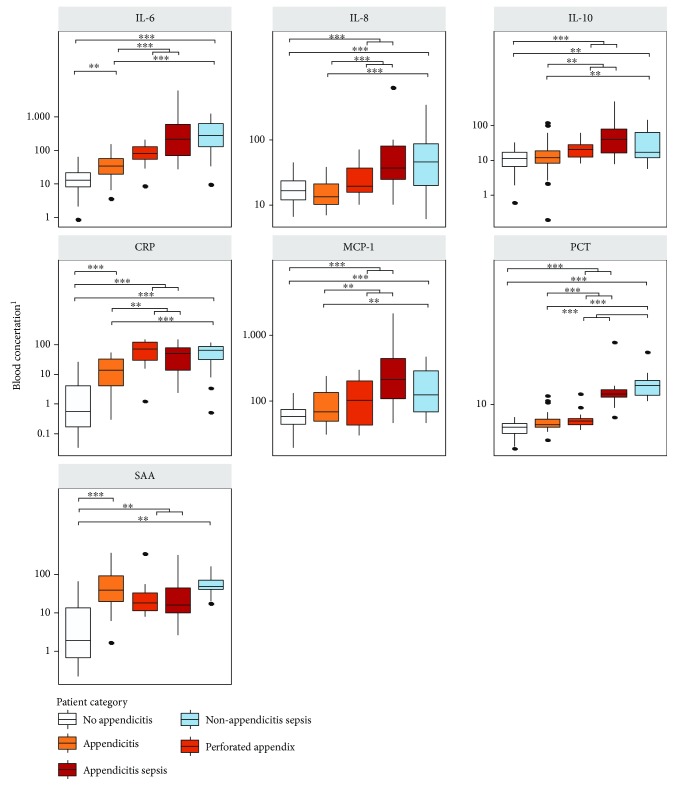
Boxplots of 7 selected cytokine concentrations in pediatric patients (*n* = 196) grouped by category and appendicitis severity. Outliers (values >95^th^ percentile) are not presented in the plots but were included in statistical comparisons conducted using multivariate normal regression followed by least-square means post hoc pairwise contrasts using the Bonferroni correction [1]. Concentrations are in ng/mL for PCT, mg/L for CRP and SAA, and pg/mL for IL-6, IL-8, IL-10, and MCP-1, ^∗^ *p* value <0.05, ^∗∗^ *p* value <0.01, and ^∗∗∗^ *p* value <0.001.

IL-6, IL-8, and PCT demonstrated the most significant difference (*p* < 0.001) between simple and complex appendicitis. SAA did not demonstrate significant differences across severity of illness (Table 2, Figure 3). Pairwise Pearson's correlation coefficients and multivariate regression model coefficients are listed in Supplementary Tables 4 and 5.

### 3.3. Expansive Inflammatory-Related Protein Mediator Profiling (Heat Mapping)

**Figure 4:**
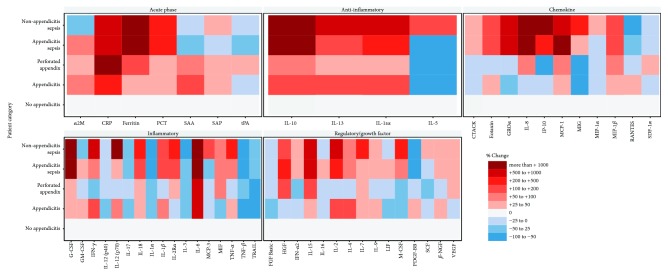
Heatmap showing relative change over mean concentration in patients without appendicitis of 54 selected cytokines grouped by patient category, appendicitis severity, and class of cytokine based on 196 pediatric patients.

Levels of acute phase reactants (7), chemokines (11), regulatory growth hormones (15), inflammatory (17), and anti-inflammatory (4) mediators were determined (mean and standard deviation for all 54 proteins found in Table S6) and compared across severity of illness; upon visual inspection, six main response patterns were observed (Figure 4).  Table S1 describes each of these inflammatory proteins.

1. Progressively higher plasma levels with increasing severity of illness: ferritin, G-CSF, IL-6, IL-15, MIP-1*β*, IL-18, MCP-1, and PCT
2. Progressive suppression of plasma levels with increasing illness severity: RANTES, IL-1*α*, TNF-*β*, and TRAIL
3. Multiphasic pattern, with initial suppression followed by progressive elevation: CTACK, MIG, IL-8, IL-2R*α*, IFN-*α*2, M-CSF, VEGF, and IP-10
4. Multiphasic pattern, with initial elevation followed by progressive suppression: RANTES, SAA, and PDGF-BB
5. Clear distinction between appendicitis (simple, perforated) and sepsis (regardless of underlying condition): PCT, CTACK, GRO*α*, G-CSF, IL-1*α*, IL-6, TRAIL, and INF-*α*2
6. Clear distinction in all appendicitis when compared to nonappendicitis sepsis: IL-5, MIP-1*α*, IL-3, IL-6, MCP-3, and IL-7

### 3.4. Protein Mediator Decision Tree Analysis

**Figure 5:**
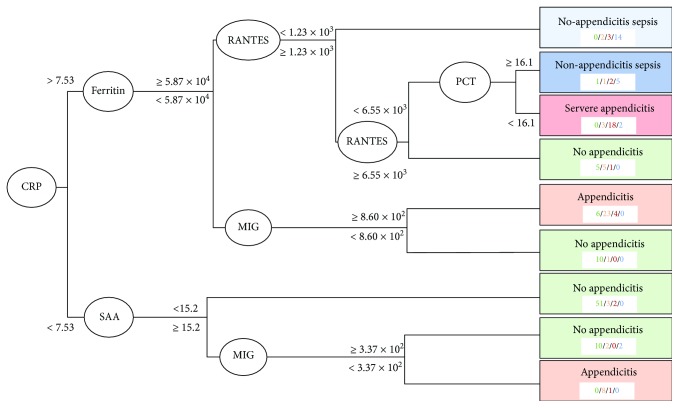
Decision tree for classifying patients into different categories or severities of appendicitis based on concentrations of 4 informative cytokines. Thresholds presented are blood concentrations of the cytokines. Concentrations are in ng/mL for PCT, mg/L for CRP and SAA, and pg/mL for all others. Numbers in the category below refer to how many individuals in each terminal node have been classified into each category: green = no appendicitis; orange = appendicitis; red = severe appendicitis; and blue = nonappendicitis sepsis.

54 protein mediators were evaluated for decision tree analysis. The final decision tree was composed of 6 biomarkers including CRP, ferritin, SAA, RANTES, MIG, and PCT (Figure 5). Operating characteristics demonstrate high levels of specificity and negative predictive values for each patient category (Table 3).

### 3.5. Assessment of Current Common Appendicitis Evaluations

Characteristics of tests commonly used in the evaluation of pediatric appendicitis (WBC, neutrophils, and PAS) are shown in Table S7.

## 4. Discussion

Current advances in, and the availability of, precision medicine technologies offer the potential to transform our understanding of the underlying physiologic responses of children with abdominal pain, providing a gateway to novel diagnostic and risk-stratification strategies. In this study, we have described the inflammatory landscape of children presenting to the ED with suspected appendicitis. Specifically, using a conventional approach based on evaluating individual bio-markers, we have demonstrated statistically significant differences in 5 previously described plasma markers (IL-6, CRP, MCP-1, PCT, and SAA) in children with appendicitis compared to those with NAAP. Furthermore, the results of our decision-tree analysis and expansive proteomic heat mapping approaches determined several potential future biomarker candidates that had not previously been identified. The identification of these inflammatory protein mediators could reveal a “fingerprint” for appendicitis and, together with future bio-technology partnership, result in a point-of-care clinical tool for timely and accurate diagnosis which is readily available/accessible across the spectrum of health care settings.

Despite being the most common atraumatic surgical emergency in the pediatric population, appendicitis continues to challenge clinicians managing children with abdominal pain, from primary care providers in rural settings to tertiary pediatric emergency subspecialists. Current diagnostic and risk stratification strategies [5] include combinations of common clinical scoring systems (Pediatric Appendicitis Score [36], Alvarado Score [37], Lintula Score [38], Appy1 [39], etc.), laboratory investigations (WBC, neutrophils +/- CPR) [1], and imaging studies (ultrasound) [40]. While often useful in the first-line work-up of a child with suspected appendicitis, these strategies remain limited because (a) children often present with atypical symptoms [41, 42], (b) clinical scoring systems and conventional laboratory investigations have suboptimal test characteristics (sensitivities and specificities in the 70-85% range) [1, 43–45], and (c) ultrasound is known to have high rates of incomplete visualization [46–49], may be painful for the child (compression on an already tender abdomen), and in females requires a full bladder (for optimal evaluation of pelvic organs) [50]. Second-line/advanced imaging techniques are limited by exposure of developing organs to unacceptable levels of ionizing radiation (computed tomography) [51] or have limited accessibility outside tertiary settings (magnetic resonance imaging). Sadly, a missed or misdiagnosis of appendicitis [52–54] can lead to adverse patient outcomes; it remains amongst the highest concerns of caregivers/parents, ranks amongst the highest pediatric emergency presentations leading to litigation [55, 56], and can result in unnecessary exposure to surgical and anesthetic intervention (negative appendectomies).

In evaluating a set of 7 previously identified inflammatory protein markers, our results demonstrated statistically significant differences in IL-6, CRP, MCP-1, PCT, and SAA. These results are consistent with prior studies [6–8, 13–15, 57]. Despite these significant differences, no single mediator demonstrates satisfactory sensitivity or specificity for the clinical identification of appendicitis. This has been a consistent problem in the application of biomarker analysis to complex disease. More recent studies have demonstrated the value in multiplex analysis of biomarkers, assessing an overall disease or inflammatory “fingerprint” over the measurement of a single mediator [30–32]. Assessment of multiple biomarkers is better able to filter out the noise originating from multiple disease etiologies (many mediators are specific to the initiating source of inflammation, independent of the actual clinical course of disease) allowing studies to focus on the handful of mediators responsible for the actual disease/tissue pathology. Importantly, inflammatory mediators do not work in isolation but rather function as an overall milieu. For example, a patient with an increase in one specific proinflammatory cytokine may have similar disease progression as a second patient who has normal levels of the proinflammatory mediator but reduced levels of an anti-inflammatory cytokine. By measuring single mediators, these overall “fingerprints” or bioprofiles are missed. Additionally, analysis of single mediators is inherently biased, focusing on the most obvious or logical targets and ignoring other mediators that may be much more biologically important but are simply not commonly studied or on the surface have no apparent mechanistic link to the condition being studied. Through the use of a large, multiplexed panel, one is better able to identify these nonobvious targets that may in fact be more informative than the common a priori selected mediators. Finally, the surge or suppression of individual biomarkers may have a temporal relationship to disease evolution that would allow a multimarker panel to identify changes across time.

The principle drawback associated with the analysis of a large array of biomediators is the need to separate the key core markers that predict disease or progression from the noise of the other background mediators. To accomplish this, we applied a machine learning approach to the overall data set to generate a decision tree. The purpose of the decision tree was to identify important markers and have a way to graphically display between-group differences rather than to develop an accurate predictive tool. The decision tree analysis identified 6 key biomarkers, as using any additional biomarkers would not have resulted in an appreciable increase in classification accuracy. Only two out of the 6 biomarkers were included in the more in-depth analysis (CRP and PCT) while the role of the other 4 (Ferritin, SAA, RANTES, and MIG) in the progression of appendicitis may be more unclear. Despite achieving the best possible fit for this current dataset, the test characteristics of the decision tree preclude its use in clinical settings; in particular, misclassification of patients with nonappendicitis sepsis and severe appendicitis as healthy would have the most severe consequences. Although this specific decision tree would require revision and validation before implementation into clinical practice as a decision tool, it can serve as a hypothesis-generating mechanism for investigating the roles of different biomarkers in pathogenesis, graphically display possible classification schemes, and demonstrate the potential importance of machine learning methods which considers multiple markers and features for disease diagnosis.

Machine learning (ML) has already successfully been applied to identify patients with chronic and long-term conditions like cancer [58] or in detecting sepsis based on continuous heart rate and blood pressure monitoring in critical care patients [59, 60]. In these cases, however, data is often available for long periods of time (cancer) or is continuously collected (critical care patients), whereas in emergency medicine collecting high volumes of sequential data is not practical or possible in short periods of time. Success with using ML methods in identifying patients with preclinical Alzheimer's and clinical cases of Alzheimer's [61–64] based on blood metabolite and protein panels could suggest the use of similar panel-based decision tools in emergency medicine. Indeed, ML methodologies are making their way into the ED [65]; they have been shown to be as accurate—or better than—certain clinical outcome prediction models for ED triage [66], predicting adverse cardiovascular outcomes [67, 68], and identifying traumatic injury requiring life-saving intervention [69, 70].

Historically, it has not been practical to conduct such a broad spectrum biomediator analysis. Measurement and characterization of more than 50 mediators in a clinical study using single analyte assessments is not feasible due to cost, time, and sample volume requirements. However, the application of multiplexed approaches, such as we have demonstrated in the current study, offers significant advantages. It is possible to generate data on scores of mediators in a cost- and time-efficient manner with very reasonable sample volumes. For the current study, less than 200 *μ*L of blood from each patient was needed; this research strategy provides significant advantages in studying even the youngest neonates in the future. Although the current approach examines a very broad array of inflammatory mediators, many of which appear to be noninformative for this study, in future studies, similar multiplex technology can be developed to focus only on the biomarkers found to identify appendicitis and stratify disease severity. This approach would allow transition from a discovery-based overall assessment of the inflammatory “landscape” to a focused, bedside diagnostic study that can accurately and specifically identify patients that would best benefit from a given treatment. Importantly, the current assessment of protein mediators can be partnered in future studies with additional panels of biomarkers (damage-associated molecular patterns [DAMPs], metabolites, transcriptomes, etc.). Although multiplex approaches provide better sensitivity and specificity than single analyte assessments, previous work has demonstrated that the integration of multiple biomarker platforms has the capacity to further enhance the accuracy of these diagnostic tests [71].

### 4.1. Limitations

One significant limitation of the current study is the turnaround time required between sample collection and determination of biomarker levels. Much of this limitation is related to the assessment of a broad array of biomarkers requiring sequential analysis of multiple assay plates. Future studies aimed at the assessment of a narrow range of useful markers will greatly streamline the assessment, allowing results to be available to the clinician within one to two hours. Further refinement and engagement with industry can enable the development of bedside, dipstick-based tests that could provide results within minutes using a single drop of blood. These features are highly relevant in busy, high-volume, high-stakes ED environments. Not only does such an approach have substantial appeal with respect to diagnostic turnaround time, but simple bedside tests do not require specialized equipment or staff expertise allowing them to be used in the smallest and most remote health care settings. Diagnosis of patients within a rural or remote setting can rapidly inform a local health care professional if transfer of the patient to the nearest surgical centre is required, reducing delays, lowering the risk of severe complication, and improving overall patient outcomes.

The dataset in our current study was limited in power for training a complex ML classification algorithm with 4 different outcome levels. In developing this kind of ML decision tool, it is important to have a diverse set of data for training models; Casanova et al. [63] have demonstrated how limited datasets can impact repeatability and reliability, which is vital in the high-stakes ED environment. External data for validating model performance is required.

## 5. Conclusions

While assessment of previously identified inflammatory plasma mediators demonstrate statistical differences in children with appendicitis when compared to those with nonappendicitis abdominal pain, analysis of individual mediators does not have sufficiently acceptable test characteristics to be used to rule in or out appendicitis. However, a precision medicine multiplex approach to evaluating the inflammatory protein mediator landscape identifies novel patterns of candidate biomarkers that could be used to identify a fingerprint of disease. Together with industry partners, point-of-care diagnostic technologies could be developed. This discovery-to-translation approach can be used across multiple acute pediatric presentations and can be modelled for future research initiatives in pediatric emergency medicine.

## Figures and Tables

**Table 1:** Characteristics of children in control, appendicitis, and sepsis cohorts.

	Nonappendicitis abdominal pain (*n* = 83)	Appendicitis (*n* = 48)	Perforated appendicitis (*n* = 17)	Appendicitis-related sepsis (*n* = 14)	Nonappendicitis sepsis (*n* = 23)
Females, *n* (%)	52 (62.7)	20 (41.7)	10 (58.8)	6 (42.9)	11 (47.8)
Age, mean (SD)	11.7 (3.6)	11.5 (3.2)	11.2 (3.6)	11.7 (3.4)	11.2 (4.1)
PAS, median (IQR)	5 (4)	7 (3)	8.5 (3)	N/A	N/A
Fever, *n* (%)	50 (60.2)	17 (35.4)	3 (17.7)	3 (21.4)	2 (8.7)
WBC, mean (SD)	10.2 (5.2)	15.6 (4.8)	17.9 (4.8)	18.1 (9.1)	15.2 (10.4)

SD: standard deviation; PAS: Pediatric Appendicitis Score; IQR: interquartile range; N/A: not available; WBC: white blood cell count.

**Table 2:** Inflammatory protein mediators and their test characteristics in children with suspected appendicitis. Area under receiver operator characteristic curve and other test characteristics calculations are based on classification of patients with nonappendicitis abdominal pain and appendicitis (nonappendicitis sepsis group is excluded).

	Nonappendicitis abdominal pain (*n* = 83)	Appendicitis (*n* = 79)	Nonappendicitis sepsis (*n* = 23)	AUROC	Cut-off (%correct)	Sensitivity: specificity	LR+ : LR-
CRP, mean (SD)^1^	7.6 (14.1)	46.6 (51.8)	70.5 (44.3)	0.8472	7.9 (78.1)	80.5 : 75.6	3.31 : 0.26
IL-6, mean (SD)^2^	28.0 (44.3)	1113.8 (6165.1)	4834.8 (20025.2)	0.8101	68.9 (69.4)	48.1 : 90.1	4.87 : 0.58
IL-8, mean (SD)^2^	24.4 (20.0)	164.5 (914.5)	385.8 (922.5)	0.5425	33.2 (59.4)	34.2 : 83.5	2.08 : 0.79
IL-10, mean (SD)^2^	19.9 (23.7)	94.9 (297.0)	271.4 (864.9)	0.6294	30.1 (62.26)	35.1 : 87.8	2.87 : 0.74
MCP-1, mean (SD ^2^)	88.3 (102.7)	361.5 (1129.2)	605.0 (1786.3)	0.6813	133.8 (68.4)	45.5 : 90.1	4.60 : 0.61
PCT, mean (SD)^3^	6.1 (2.3)	10.0 (9.2)	33.1 (52.0)	0.7382	6.5 (69.2)	57.7 : 80.8	3.00 : 0.52
SAA, mean (SD)^1^	122.7 (254.5)	248.3 (377.0)	92.5 (83.5)	0.7514	73.2 (73.6)	93.6 : 53.1	2.05 : 0.12

CRP: c-reactive protein; IL: interleukin; MCP: monocyte chemoattractant protein; PCT: procalcitonin; SAA: serum amyloid A; AUROC: area under receiver operating characteristic; LR: likelihood ratio. ^1^Concentrations measured in mg/L, ^2^concentrations measured in pg/mL, ^3^concentrations measured in ng/mL.

**Table 3:** Operating characteristics (95% confidence interval) of patient categorization based on the decision tree.

Patient category	Sensitivity	Specificity	PPV	NPV
Nonappendicitis abdominal pain	0.916 (0.834-0.965)	0.843 (0.758-0.908)	0.826 (0.733-0.897)	0.925 (0.851-0.969)
Simple appendicitis	0.646 (0.495-0.778)	0.920 (0.862-0.960)	0.738 (0.580-0.861)	0.882 (0.818-0.930)
Severe appendicitis	0.581 (0.391-0.755)	0.968 (0.926-0.989)	0.783 (0.563-0.925)	0.920 (0.867-0.957)
Nonappendicitis sepsis	0.826 (0.612-0.950)	0.945 (0.898-0.974)	0.679 (0.476-0.841)	0.975 (0.936-0.993)
Nonappendicitis abdominal pain	0.916 (0.834-0.965)	0.860 (0.776-0.921)	0.844 (0.753-0.912)	0.925 (0.851-0.969)
Appendicitis	0.722 (0.609-0.817)	0.925 (0.857-0.967)	0.877 (0.772-0.945)	0.817 (0.736-0.881)
Nonappendicitis sepsis	0.826 (0.612-0.950)	0.944 (0.897-0.974)	0.679 (0.476-0.841)	0.975 (0.936-0.993)

## Data Availability

The data used to support the findings of this study are available from the corresponding author upon request.

## Supplementary Materials

Supplementary Materials Table S1: Luminex multiplex assays of inflammatory protein mediators. Table S2: pairwise Pearson's correlation coefficient computed between cytokine concentrations of pediatric patients stratified by outcome category. Table S3: pairwise Pearson's correlation coefficient computed between cytokine concentrations of pediatric patients stratified by outcome category and severity of appendicitis. Table S4: model coefficients for the multivariate normal regression fit assessing differences between cytokine concentrations in pediatric patients (*n* = 185) of different categories. Includes an adjustment for age and sex of patient. Table S5: model coefficients for the multivariate normal regression fit assessing differences between cytokine concentrations in pediatric patients of different categories and severity of appendicitis. Includes an adjustment for age and sex of patient. Table S6: mean and standard deviation of 54 protein mediators in children with suspected appendicitis. Table S7: test characteristics of current “gold standard” evaluations in children with suspected appendicitis. Figure S1: boxplots of 7 selected cytokine concentrations in pediatric patients grouped by category, with outliers (values >95th percentile) included. Figure S2: boxplots of 7 selected cytokine concentrations in pediatric patients grouped by category and appendicitis severity, with outliers (values >95th percentile) included.
