# Supplementary material for: Cytokines and Chemokines in Pediatric Appendicitis: A Multiplex Analysis of Inflammatory Protein Mediators
Source: Mediators Inflamm. 2019 Feb 21;2019:2359681. doi: 10.1155/2019/2359681 (PMC6409077; doi:10.1155/2019/2359681)

Supplementary Materials For Manuscript: Cytokines and chemokines in pediatric appendicitis: a multiplex analysis of inflammatory protein mediators.

Author List: Ali Naqvi BScH[1,2], Graham C Thompson MD[3,4], Ari Joffe MD[5], Jaime Blackwood MD[3], Dori-Ann Martin BScN[3], Mary Brindle MD[6], Herman W. Barkema DVM, PhD[2], Craig N. Jenne PhD[7,8].

Supplementary tables and figures in this file include:

Table S1. Luminex Multiplex Assays of Inflammatory Protein Mediators
Table S2. Pairwise Pearson's correlation coefficient computed between cytokine concentrations of pediatric patients stratified by outcome category.
Table S3. Pairwise Pearson's correlation coefficient computed between cytokine concentrations of pediatric patients stratified by outcome category and severity of appendicitis.
Table S4. Model coefficients for the multivariate normal regression fit assessing differences between cytokine concentrations in pediatric patients (n=185) of different categories. Includes an adjustment for age and sex of patient.
Table S5. Model coefficients for the multivariate normal regression fit assessing differences between cytokine concentrations in pediatric patients of different categories and severity of appendicitis. Includes an adjustment for age and sex of patient.
Table S6. Mean and standard deviation of 54 protein mediators in children with suspected appendicitis.
Table S7. Test characteristics of current "gold standard" evaluations in children with suspected appendicitis.
Figure S1. Boxplots of 7 selected cytokine concentrations in pediatric patients grouped by category, with outliers (values > 95th percentile) included.
Figure S2. Boxplots of 7 selected cytokine concentrations in pediatric patients grouped by category and appendicitis severity, with outliers (values > 95th percentile) included.

Table S1. Luminex Multiplex Assays of Inflammatory Protein Mediators.

Assay Kits (Bio-Rad Laboratories)	Inflammatory Protein Mediators
Bio-Plex Pro™ Human Cytokine 21-plex (#MG0005KMII)	Interleukin (IL)-1α, IL-2Rα, IL-3, IL-12 (p40), IL-16, IL18, cutaneous T-cell attracting chemokine (CTACK), growth related oncogene (Gro)-α, hepatocyte growth factor (HGF), interferon (IFN)-α2, leukemia inhibitory factor (LIF), monocyte chemotactic protein (MCP)-3, monocyte colony stimulating factor (M-CSF), macrophage migration inhibitory factor (MIF), monokine induced by gamma interferon (MIG), β-nerve growth factor (NGF), stem cell factor (SCF), stem cell growth factor (SCGF)-β, stromal cell-derived factor (SDF)-1α, tumor necrosis factor (TNF)-β, TNF-related apoptosis-inducing ligand (TRAIL).
Bio-Plex Pro™ Human Cytokine 27-plex (#M500KCAF0Y)	Fibroblast growth factor basic (FGF2), eotaxin, granulocyte colony-stimulating factor (G-CSF), granulocyte-macrophage colony-stimulating factor (GM-CSF), IFN-γ, IL-1β, IL-1ra, IL-2, IL-4, IL-5, IL-6, IL-7, IL-8, IL-9, IL-10, IL-12 (p70), IL-13, IL-15, IL-17, Interferon gamma-induced protein (IP)-10, monocyte chemotactic protein (MCP)-1 , macrophage inflammatory protein (MIP)-1α and -1β, platelet-derived growth factor (PDGF)-BB, regulated on activation normal T-cell expressed and secreted (RANTES), tumour necrosis factor (TNF)-α, vascular endothelial growth factor (VEGF).
Bio-Plex Pro™ Human Acute Phase 5 + 4-plex Panel Complete (#171A4S07M)	Ferritin, fibrinogen, procalcitonin (PCT), serum amyloid A (SAA), tissue plasminogen activator (TPA), α-2-macroglobin (α2M), C-reactive protein (CRP), haptoglobin, serum amyloid P (SAP).

Table S2. Pairwise Pearson's correlation coefficient computed between cytokine concentrations of pediatric patients stratified by outcome category. Top-half of correlation matrix is omitted as it mirrors values in the lower half.

Patient Category (n)	Cytokine	IL-6	IL-8	IL-10	CRP	MCP-1	PCT	SAA
No appendicitis (83)	IL-6	1.000						
	IL-8	0.373	1.000					
	IL-10	0.465	0.434	1.000				
	CRP	0.415	0.223	0.057	1.000			
	MCP-1	0.385	0.334	0.343	0.165	1.000		
	PCT	0.414	-0.010	0.184	0.429	0.202	1.000	
	SAA	0.407	0.199	0.129	0.855	0.164	0.430	1.000
Appendicitis (79)	Cytokine	IL-6	IL-8	IL-10	CRP	MCP-1	PCT	SAA
	IL-6	1.000						
	IL-8	0.817	1.000					
	IL-10	0.590	0.595	1.000				
	CRP	0.371	0.312	0.162	1.000			
	MCP-1	0.746	0.766	0.459	0.266	1.000		
	PCT	0.746	0.745	0.418	0.277	0.746	1.000	
	SAA	-0.255	-0.233	-0.242	0.010	-0.001	-0.143	1.000
Non-appendicitis sepsis (23)	Cytokine	IL-6	IL-8	IL-10	CRP	MCP-1	PCT	SAA
	IL-6	1.000						
	IL-8	0.703	1.000					
	IL-10	0.729	0.603	1.000				
	CRP	-0.208	0.000	-0.115	1.000			
	MCP-1	0.743	0.810	0.737	-0.022	1.000		
	PCT	0.688	0.605	0.711	0.041	0.712	1.000	
	SAA	0.112	0.146	0.318	-0.100	0.140	0.078	1.000

Table S3. Pairwise Pearson's correlation coefficient computed between cytokine concentrations of pediatric patients stratified by outcome category and severity of appendicitis. Top-half of correlation matrix is omitted as it mirrors values in the lower half.

Patient Category (n)	Cytokine	IL-6	IL-8	IL-10	CRP	MCP-1	PCT	SAA
No appendicitis (83)	IL-6	1.000						
	IL-8	0.373	1.000					
	IL-10	0.465	0.434	1.000				
	CRP	0.415	0.223	0.057	1.000			
	MCP-1	0.385	0.334	0.343	0.165	1.000		
	PCT	0.414	-0.010	0.184	0.429	0.202	1.000	
	SAA	0.407	0.199	0.129	0.855	0.164	0.430	1.000
Appendicitis (48)	Cytokine	IL-6	IL-8	IL-10	CRP	MCP-1	PCT	SAA
	IL-6	1.000						
	IL-8	0.695	1.000					
	IL-10	0.347	0.338	1.000				
	CRP	0.255	0.406	-0.021	1.000			
	MCP-1	0.598	0.457	-0.019	0.297	1.000		
	PCT	0.482	0.292	-0.122	0.234	0.594	1.000	
	SAA	0.037	0.133	-0.041	0.255	0.318	0.288	1.000
Severe appendicitis (31)	Cytokine	IL-6	IL-8	IL-10	CRP	MCP-1	PCT	SAA
	IL-6	1.000						
	IL-8	0.841	1.000					
	IL-10	0.702	0.710	1.000				
	CRP	0.187	0.016	0.042	1.000			
	MCP-1	0.773	0.821	0.722	0.014	1.000		
	PCT	0.815	0.826	0.687	0.036	0.754	1.000	
	SAA	-0.370	-0.326	-0.346	-0.028	-0.067	-0.306	1.000
Non-appendicitis sepsis (23)	Cytokine	IL-6	IL-8	IL-10	CRP	MCP-1	PCT	SAA
	IL-6	1.000						
	IL-8	0.703	1.000					
	IL-10	0.729	0.603	1.000				
	CRP	-0.208	0.000	-0.115	1.000			
	MCP-1	0.743	0.810	0.737	-0.022	1.000		
	PCT	0.688	0.605	0.711	0.041	0.712	1.000	
	SAA	0.112	0.146	0.318	-0.100	0.140	0.078	1.000

Table S4. Model coefficients for the multivariate normal regression fit assessing differences between cytokine concentrations in pediatric patients of different categories. Includes an adjustment for age and sex of patient.

Cytokine	Coefficient	Estimate	Standard Error	*P*-value
IL-6	Intercept†	2.720	0.207	< 0.001
	Appendicitis	1.673	0.256	< 0.001
	Non-appendicitis sepsis	3.363	0.385	< 0.001
	Age	-0.065	0.033	0.050
	Sex	0.137	0.240	0.569
IL-8	Intercept†	2.965	0.153	< 0.001
	Appendicitis	0.321	0.188	0.091
	Non-appendicitis sepsis	1.451	0.284	< 0.001
	Age	-0.033	0.024	0.172
	Sex	0.055	0.177	0.758
IL-10	Intercept†	2.611	0.186	< 0.001
	Appendicitis	0.547	0.230	0.019
	Non-appendicitis sepsis	1.250	0.346	< 0.001
	Age	-0.059	0.030	0.049
	Sex	-0.067	0.216	0.756
CRP	Intercept†	7.321	0.246	< 0.001
	Appendicitis	2.851	0.303	< 0.001
	Non-appendicitis sepsis	3.555	0.456	< 0.001
	Age	-0.115	0.039	0.004
	Sex	-0.243	0.284	0.394
MCP-1	Intercept†	4.125	0.137	< 0.001
	Appendicitis	0.760	0.170	< 0.001
	Non-appendicitis sepsis	1.290	0.255	< 0.001
	Age	0.009	0.022	0.675
	Sex	0.030	0.159	0.850
PCT	Intercept†	8.619	0.070	< 0.001
	Appendicitis	0.383	0.087	< 0.001
	Non-appendicitis sepsis	1.326	0.131	< 0.001
	Age	0.002	0.011	0.860
	Sex	0.107	0.082	0.190
SAA	Intercept†	9.048	0.296	< 0.001
	Appendicitis	2.048	0.366	< 0.001
	Non-appendicitis sepsis	1.956	0.550	< 0.001
	Age	-0.040	0.047	0.397
	Sex	0.171	0.343	0.619

†Intercept refers to reference group against which the coefficients are being compared: females, of the mean age (11.16yr) with no appendicitis.

Table S5. Model coefficients for the multivariate normal regression fit assessing differences between cytokine concentrations in pediatric patients of different categories and severity of appendicitis. Includes an adjustment for age and sex of patient.

Cytokine	Coefficient	Estimate	Standard Error	*P*-value
IL-6	Intercept†	2.691	0.194	< 0.001
	Appendicitis	0.981	0.281	< 0.001
	Severe appendicitis	2.557	0.305	< 0.001
	Non-appendicitis sepsis	3.359	0.360	< 0.001
	Age	-0.063	0.031	0.044
	Sex	0.206	0.225	0.362
IL-8	Intercept†	2.945	0.145	< 0.001
	Appendicitis	-0.132	0.211	0.532
	Severe appendicitis	0.898	0.228	< 0.001
	Non-appendicitis sepsis	1.449	0.270	< 0.001
	Age	-0.032	0.023	0.169
	Sex	0.100	0.168	0.555
IL-10	Intercept†	2.591	0.180	< 0.001
	Appendicitis	0.072	0.261	0.782
	Severe appendicitis	1.152	0.282	< 0.001
	Non-appendicitis sepsis	1.247	0.334	< 0.001
	Age	-0.057	0.029	0.047
	Sex	-0.020	0.209	0.925
CRP	Intercept†	7.295	0.238	< 0.001
	Appendicitis	2.242	0.344	< 0.001
	Severe appendicitis	3.627	0.373	< 0.001
	Non-appendicitis sepsis	3.552	0.441	< 0.001
	Age	-0.113	0.038	0.003
	Sex	-0.182	0.275	0.508
MCP-1	Intercept†	4.110	0.133	< 0.001
	Appendicitis	0.413	0.192	0.033
	Severe appendicitis	1.203	0.208	< 0.001
	Non-appendicitis sepsis	1.288	0.246	< 0.001
	Age	0.010	0.021	0.626
	Sex	0.065	0.154	0.675
PCT	Intercept†	8.611	0.067	< 0.001
	Appendicitis	0.185	0.098	0.060
	Severe appendicitis	0.637	0.106	< 0.001
	Non-appendicitis sepsis	1.325	0.125	< 0.001
	Age	0.003	0.011	0.807
	Sex	0.127	0.078	0.106
SAA	Intercept†	9.065	0.294	< 0.001
	Appendicitis	2.443	0.427	< 0.001
	Severe appendicitis	1.543	0.462	0.001
	Non-appendicitis sepsis	1.958	0.547	< 0.001
	Age	-0.041	0.047	0.378
	Sex	0.132	0.341	0.700

†Intercept refers to reference group against which the coefficients are being compared: females, of the mean age (11.16yr) without appendicitis.

Table S6. Mean and standard deviation of 54 protein mediators in children with suspected appendicitis. All concentrations measured in pg/mL unless otherwise marked.

Category	Protein	Non-appendicitis Abdominal Pain (n=84)	Appendicitis (n=50)	Perforated Appendicitis (n=17)	Appendicitis-related Sepsis (n=14)	Non-appendicitis Sepsis (n=31)
**Inflammatory**	IL-1β	3.06 (11.49)	4.76 (11.49)	2.56 (1.79)	7.34 (12.36)	8.71 (27.24)
	IL-1rα	28.04 (568.32)	197.43 (568.32)	222.5 (393.41)	5337.7 (14259.47)	4834.78 (20025.24)
	IL-2	31.03 (88.38)	49.34 (88.38)	26.98 (33.72)	43 (51.16)	406.08 (1142.31)
	IL-4	91.25 (57.85)	65.49 (57.85)	82.1 (97.58)	61.74 (57.19)	51.47 (96.42)
	IL-5	227.46 (297.25)	262.06 (297.25)	296.43 (311.33)	11003.57 (30278.86)	5683.09 (17321.13)
	IL-6	56.7 (79.56)	75 (79.56)	54.75 (25.6)	61.62 (67.69)	39.48 (65.92)
	IL-7	102.96 (673.69)	231.54 (673.69)	64.9 (56.05)	156.45 (193.6)	624.83 (2392.47)
	IL-8	37.94 (135.31)	56.11 (135.31)	35.28 (45.45)	59.48 (66.69)	149.3 (498.36)
	IL-9	15.53 (9.84)	10.73 (9.84)	11.59 (11.65)	2.99 (4.36)	1.08 (1.99)
	IL-10	224.47 (156.02)	194.67 (156.02)	256.22 (175.56)	623.3 (624.74)	827.08 (1007.93)
	IL-12(p70)	1063.25 (706.37)	701.92 (706.37)	729.83 (587.9)	657.35 (1015.87)	329.39 (647.94)
	IL-13	1251.38 (735.85)	854.78 (735.85)	1038.81 (1099.58)	950.69 (1426.8)	1021.15 (2148.51)
	IL-15	83.92 (64.36)	84.13 (64.36)	87.19 (39.23)	193.86 (131.86)	392.61 (363.08)
	IL-17	96.35 (123.9)	86.57 (123.9)	75.86 (120.25)	49.47 (77.62)	209.94 (698.44)
	Eotaxin	281.31 (185.22)	179.89 (185.22)	497.93 (857.34)	478.17 (400.51)	462.48 (382.13)
	FGF Basic	14.53 (8.66)	6.22 (8.66)	7.61 (9.3)	4.11 (5.98)	2.14 (3.08)
	G-CSF	167.48 (57.92)	80.78 (57.92)	61.7 (74.01)	93.13 (90)	113.04 (186.99)
**Anti-Inflammatory**	GM-CSF	336.94 (1826.48)	839.12 (1826.48)	404.26 (481.62)	1867.09 (2389.17)	3001.06 (8695.13)
	IFN-γ	7.94 (3.23)	2.13 (3.23)	1.49 (1.46)	1.48 (0.91)	18.16 (56.39)
	IP-10	19.91 (121.03)	49.54 (121.03)	36.36 (36.46)	314.94 (632)	271.41 (864.88)
	MCP-1	5.27 (23.52)	10.62 (23.52)	6.11 (10.2)	11.61 (10.3)	37.12 (123.21)
**Regulatory**	MIP-1α	12.7 (65.61)	31.24 (65.61)	12.58 (19.45)	47.67 (102.83)	94.73 (231.41)
	PDGF-BB	1.61 (8.69)	3.4 (8.69)	1.58 (1.24)	2.7 (2.08)	2.92 (3.97)
	MIP-1β	10.39 (27.34)	14.56 (27.34)	12.24 (14.45)	12.65 (12.49)	54.84 (176.83)
	RANTES	29.95 (55.03)	36.14 (55.03)	28.78 (30.53)	31.49 (17.84)	42.14 (52.96)
	TNF-α	4.73 (10.88)	7.05 (10.88)	13.19 (16.15)	37.3 (56.77)	29.62 (56.01)
	VEGF	68.85 (27.55)	48.64 (27.55)	59.3 (42.84)	58.73 (32.19)	54.81 (47.41)
	IL-1α	268.51 (299.95)	279.49 (299.95)	233.41 (224.9)	111.33 (62.52)	120.42 (118.88)
	IL-2Rα	52.61 (24.63)	49.84 (24.63)	55.12 (36.56)	75.87 (50.75)	76.28 (114.11)
	IL-3	475.03 (224.47)	411.14 (224.47)	450.5 (275.41)	511.33 (369.03)	457.29 (325.58)
	IL-12(p40)	356.97 (274.26)	354.86 (274.26)	789.58 (743.33)	1607.22 (1404.51)	928.98 (664.94)
	IL-16	79.37 (69.76)	71.13 (69.76)	57.59 (40.47)	83.69 (39.06)	97.06 (77.22)
	IL-18	30.49 (27.41)	22.48 (27.41)	25.52 (28.38)	30.5 (21.57)	23.9 (53.24)
	CTACK	35.4 (20.02)	23.73 (20.02)	24.8 (20.52)	67.39 (70.74)	124.6 (337.73)
	GROα	3.9 (12.21)	5.52 (12.21)	3.33 (3.09)	5.63 (5.27)	5.48 (2.8)
	HGF	165.64 (84.54)	124.81 (84.54)	114.5 (58.27)	159.4 (118.39)	210.09 (181.72)
**Chemokine**	IFN-α2	24.37 (16.87)	21.41 (16.87)	43.64 (48.9)	770.92 (2081.97)	385.83 (922.46)
	LIF	40.49 (34.11)	46.5 (34.11)	32.01 (30.25)	100.36 (94.67)	116.13 (225.43)
	MCP-3	553.53 (864.12)	484.35 (864.12)	206.67 (165.27)	2891.13 (5579.93)	6803.96 (23573.25)
	M-CSF	88.32 (115.79)	129.72 (115.79)	164.76 (130.98)	1361.91 (2463.48)	604.99 (1786.25)
	MIF	4.68 (1.75)	3.98 (1.75)	4.29 (2.36)	4.64 (1.7)	4.89 (5.17)

	MIG	85.78 (64.83)	105.09 (64.83)	148.83 (174.25)	185.38 (118.52)	244.06 (558.94)
	β-NGF	5441.47 (3523.72)	5715.89 (3523.72)	4976.66 (3378.26)	3339.55 (3587.5)	1781.21 (1824.66)
	SCF	1058.86 (327.13)	904.19 (327.13)	977.03 (473.31)	1191.78 (666.65)	1329.83 (964.45)
	SDF-1α	92.66 (49.2)	64.3 (49.2)	88.23 (77.32)	867.44 (2477.29)	573.5 (1616.44)
	TNF-β	1958.52 (2540.74)	764.73 (2540.74)	602.98 (1251.25)	2299.7 (3371.95)	6584.13 (18715.15)
	TRAIL	321.12 (86.87)	272.81 (86.87)	329.56 (171.26)	256.62 (114.3)	260.62 (213.53)
**Acute Phase**	PCT[1]	6.09 (30.60)	7.35 (30.60)	8.12 (4.03)	20.98 (17.00)	33.06 (51.93)
	Ferritin	35519.11 (30061.62)	52622.23 (30061.62)	79630.09 (63234.64)	547857.19 (1022728.32)	444619.05 (979265.21)
	tPA	4550.68 (1837.21)	4252.04 (1837.21)	4697.83 (1337.32)	2347.36 (1086.55)	3615.36 (1475.96)
	SAA[2]	122.73 (435.79)	312.36 (435.79)	200.68 (296.19)	90.96 (132.13)	92.52 (83.47)
	α2M[1]	1680543.36 (3497764.31)	2926176.71 (3497764.31)	1953556.09 (1621340.26)	3034418.33 (3917537.66)	1101392.78 (422610.36)
	CRP[2]	7.62 (21.80)	23.41 (21.80)	91.82 (65.34)	72.97 (62.31)	70.58 (44.28)
	SAP[1]	25574.33 (16462.68)	26301.23 (16462.68)	27670.08 (12649.25)	24577.78 (14834.48)	32841.91 (13573.2)

[1]Concentrations measured in ng/mL

[2]Concentrations measured in mg/L

Table S7. Test characteristics of current "gold standard" evaluations in children with suspected appendicitis.

	Cut-off Value	Sensitivity	Specificity	Correctly Classified
**WBC** (x10^3/μL)	>10	88.9%	58.4%	72.1%
	>12	81.0%	71.4%	75.7%
	>15	61.9%	80.5%	72.1%
**Neutrophils** (x10^3/μL)	>8	85.7%	74.3%	78.6%
	>10	78.6%	80.0%	79.5%
**PAS**	≥7	73.5%	71.7%	72.3%

WBC – white blood cell count; PAS – pediatric appendicitis score.

Figure S1. Boxplots of 7 selected cytokine concentrations in pediatric patients (n=196) grouped by category, with outliers (values > 95th percentile) included.

[1] Concentrations are in ng/mL for PCT; mg/L for CRP, and SAA; pg/mL for IL-6, IL-8, IL-10, and MCP-1

* *P*-value < 0.05; ** *P*-value < 0.01; *** *P*-value < 0.001

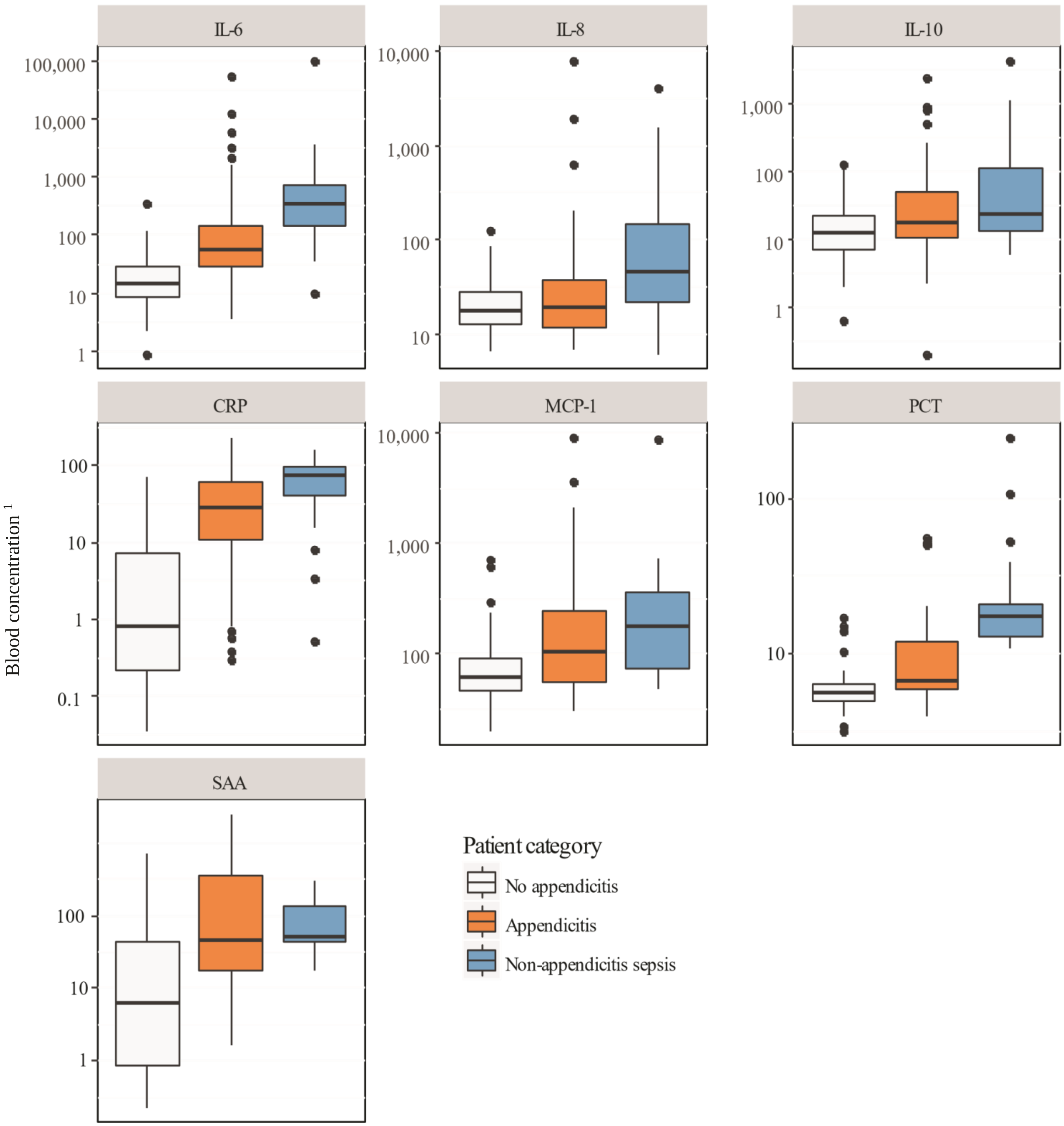

Figure S2. Boxplots of 7 selected cytokine concentrations in pediatric patients (n=196) grouped by category and appendicitis severity, with outliers (values > 95th percentile) included.
[1] Concentrations are in ng/mL for PCT; mg/L for CRP, and SAA; pg/mL for IL-6, IL-8, IL-10, and MCP-1
* *P*-value < 0.05; ** *P*-value < 0.01; *** *P*-value < 0.001

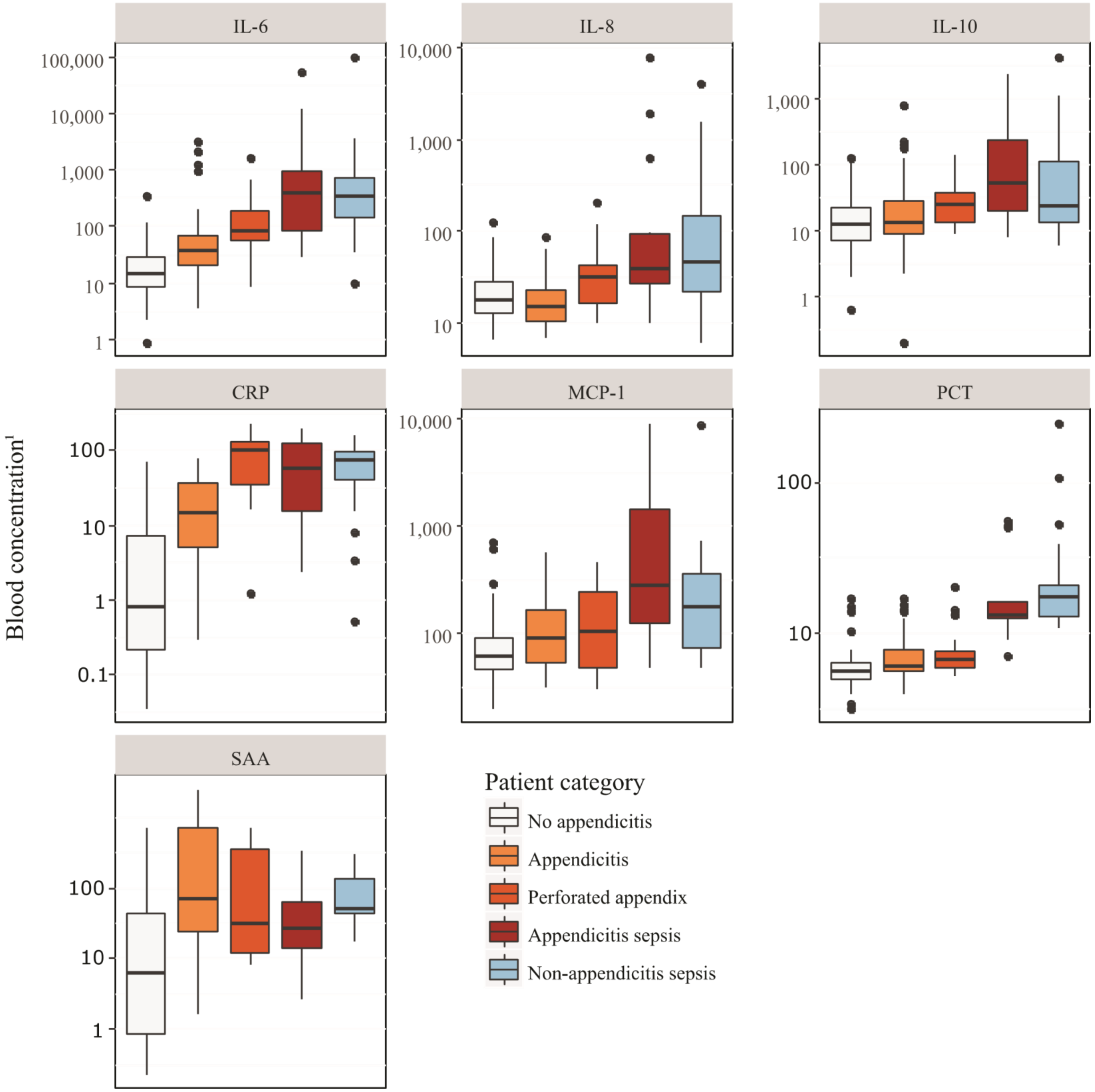

Supplement: Supplementary Materials — Table S1: Luminex multiplex assays of inflammatory protein mediators. Table S2: pairwise Pearson's correlation coefficient computed between cytokine concentrations of pediatric patients stratified by outcome category. Table S3: pairwise Pearson's correlation coefficient computed between cytokine concentrations of pediatric patients stratified by outcome category and severity of appendicitis. Table S4: model coefficients for the multivariate normal regression fit assessing differences between cytokine concentrations in pediatric patients (n = 185) of different categories. Includes an adjustment for age and sex of patient. Table S5: model coefficients for the multivariate normal regression fit assessing differences between cytokine concentrations in pediatric patients of different categories and severity of appendicitis. Includes an adjustment for age and sex of patient. Table S6: mean and standard deviation of 54 protein mediators in children with suspected appendicitis. Table S7: test characteristics of current “gold standard” evaluations in children with suspected appendicitis. Figure S1: boxplots of 7 selected cytokine concentrations in pediatric patients grouped by category, with outliers (values >95th percentile) included. Figure S2: boxplots of 7 selected cytokine concentrations in pediatric patients grouped by category and appendicitis severity, with outliers (values >95th percentile) included. [file 2359681.f1.pdf]
